# A statistical mechanics investigation of unfolded protein response across organisms

**DOI:** 10.1038/s41598-024-79086-8

**Published:** 2024-11-12

**Authors:** Nicole Luchetti, Keith M. Smith, Margherita A. G. Matarrese, Alessandro Loppini, Simonetta Filippi, Letizia Chiodo

**Affiliations:** 1 Department of Engineering, Università Campus Bio-Medico di Roma, Via Álvaro del Portillo 21, Rome, 00128 Italy; 2 Center for Life Nano- & Neuro-Science, Italian Institute of Technology, Viale Regina Elena 291, Rome, 00161 Italy; 3 Computer and Information Sciences, University of Strathclyde, 26 Richmond Street, Glasgow, G1 1XH United Kingdom; 4 Department of Medicine and Surgery, Università Campus Bio-Medico di Roma, Via Álvaro del Portillo 21, Rome, 00128 Italy; 5 National Institute of Optics, National Research Council, Largo Enrico Fermi 6, Florence, 50125 Italy; 6 International Center for Relativistic Astrophysics Network, Piazza della Repubblica 10, Pescara, 65122 Italy

**Keywords:** Endoplasmic reticulum stress, Complex networks, Protein-protein interactions, Network analysis, Computational biophysics, Control theory, Robustness, Statistical methods

## Abstract

Living systems rely on coordinated molecular interactions, especially those related to gene expression and protein activity. The Unfolded Protein Response is a crucial mechanism in eukaryotic cells, activated when unfolded proteins exceed a critical threshold. It maintains cell homeostasis by enhancing protein folding, initiating quality control, and activating degradation pathways when damage is irreversible. This response functions as a dynamic signaling network, with proteins as nodes and their interactions as edges. We analyze these protein-protein networks across different organisms to understand their intricate intra-cellular interactions and behaviors. In this work, analyzing twelve organisms, we assess how fundamental measures in network theory can individuate seed proteins and specific pathways across organisms. We employ network robustness to evaluate and compare the strength of the investigated protein-protein interaction networks, and the structural controllability of complex networks to find and compare the sets of driver nodes necessary to control the overall networks. We find that network measures are related to phylogenetics, and advanced network methods can identify main pathways of significance in the complete Unfolded Protein Response mechanism.

## Introduction

**Fig. 1:**
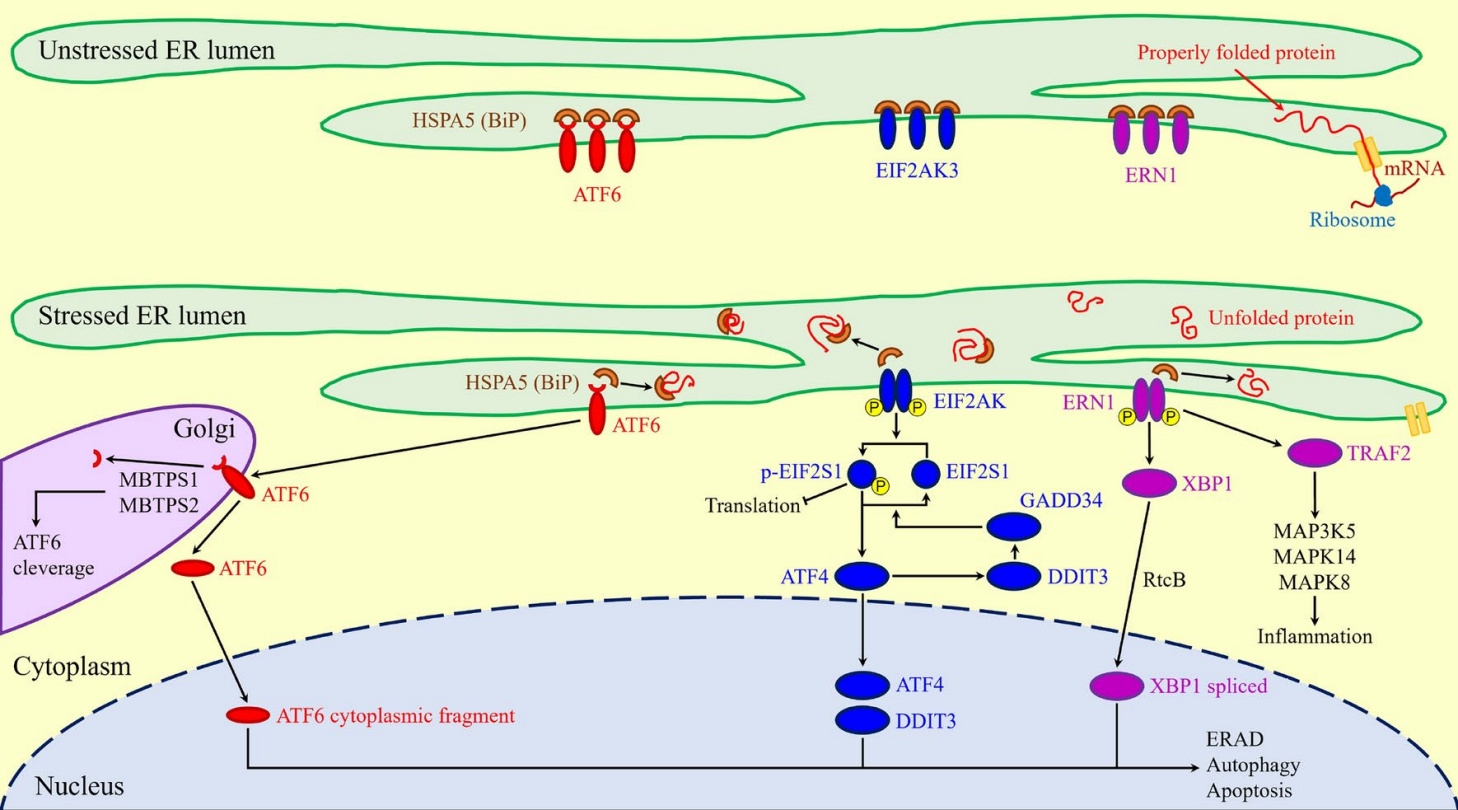
Visual representation of main UPR signaling pathways during ER stress in vertebrates. The cellular process of post-translational modification and protein folding becomes strained, leading to the buildup of non-properly folded proteins. This accumulation can eventually trigger cell death. The cell initiates a cascade of signaling pathways to counteract ER stress and regain homeostasis. These pathways enhance the production of proteins involved in proper protein folding or facilitate the removal of misfolded proteins through Endoplasmic-reticulum-associated protein degradation (ERAD). (Reproduction under the Creative Commons Attribution License).

The Unfolded Protein Response (UPR)^1^ is a mechanism adopted by cells to maintain homeostasis within the endoplasmic reticulum (ER) compartment in response to an accumulation of unfolded or improperly folded proteins (Fig. 1)^2–4^. When protein concentration exceeds physiological levels, pro-survival mechanisms are activated to restore the balance between folded and unfolded proteins^5–7^. The heat shock protein family A member 5 (HSPA5), also known as binding immunoglobulin protein (BiP)^8^, is a key promotor of the UPR, activating three stress sensors in the ER: the activating transcription factor 6 (ATF6), the endoplasmic reticulum to nucleus signaling 1 (ERN1), and the eukaryotic translation initiation factor 2 alpha kinase 3 (EIF2AK3), respectively^9–11^. If the adaptive UPR response fails, other pathways are activated, leading to apoptosis and autophagy^6,12^. This mechanism is essential for cell survival in mammals^1,13,14^and is strongly preserved across various organisms, from vertebrates to yeasts and worms^15–17^, as well as in fungi^18^and plants^19,20^.

The advancement of network theory has significantly contributed to studying biological networks, particularly protein-protein interaction (PPI) networks^21–24^. Indeed, by conducting network modeling and topological analysis, researchers can gain insights into genes and proteins involved in various biological functions and disease mechanisms^25–27^The UPR pathway can be described as a PPI network^28–30^and analyzed using complex network tools^31–36^. Protein interaction information is stored in public databases^37–40^, and obtained via direct and indirect information (i.e., from experimental Y2H test and homology). Classic measures in network theory, whose definition is briefly reported in Table 1, provide valuable insights into network structure and function. Still, they do not adequately address the dynamic aspects of network behavior and vulnerability to disruptions. Therefore, structural controllability^41,42^and network robustness^43–45^ can be used to identify driver nodes and exploit whether the network withstands failures or attacks.

Here we study the properties of the ER stress response in twelve different organisms, to determine if network analysis methods can provide insight into characteristics of PPI networks. Specifically, we want to identify and analyze the factors that impact the “strength” of various UPR networks and their resistance to potential alterations. This includes looking at random-based and various metric-based attack strategies and identifying similarities between different organism models. Our findings indicate that the several adopted methods can uncover different network characteristics, such as phylogenetic similarities^1^, distinguishing mammals and their animal models, and identifying relevant molecular pathways within the UPR mechanism across organisms. Thus, we hypothesize that these network methods can be widely applied to characterize unknown PPI networks in silico.

**Table 1:** Definition of quantities used to describe PPI networks.

Term/metric	Definition
Barycenter	The node with the lowest value of eccentricity (for us, the absolute center of the networks)
Betweenness centrality^46^	Measure of how often a node occurs on the shortest paths between other nodes
Closeness centrality	$C(x)=(\sum _y d(x,y))^{-1}$ is a measure of how close a node is to all other nodes in the network
Clustering coefficient	Proportion of edges between the nodes within the i^th^ neighborhood divided by the number of links that could exist between them
Average clustering coefficient	$CC = (\sum _i CC_i/n)$ is the arithmetic mean of the clustering coefficient of all the nodes
Density^47^	$D=2M/[N \cdot (N-1)]$, where *M* is the total number of connections in an *N* nodes network
Degree	Number of edges of one node
Average Degree	Arithmetic mean of degrees of all network nodes
Diameter	It is defined as the eccentricity of a node with the maximum distance to the other nodes
Edges	Physical or functional connections between pairs of proteins
Modules or Communities	Sub networks that include a high number of inside-sub network edges and a low number of between-sub network edges
Modularity	A measure of network tendencies to divide in communities
Nodes	Proteins composing the network
Shortest path length	Number of edges needed to connect every pair of nodes through their shortest path

## Results

The results are presented in three subsections, each elucidating the potential descriptive and predictive power of the methods employed. These methods allow the association of network properties with phylogenetic analogies and assist in identifying biological weaknesses through advanced network descriptors. These subsections correspond to the methods tested in this study: i) standard network descriptors^48^, as well as topological analysis^49^, ii) robustness^43–45^, and iii) structural controllability^41,42^. The methodological pipeline starts by establishing a native UPR model network for each organism, as detailed in Table 2, utilizing PPI data sourced from public databases. We then create configuration models by randomizing connections while preserving the same number of connections per protein. Well-established network theory measures and advanced network methods are then applied to evaluate the networks for each organism and model. In Table 2 we also report the average value of network features for each native model.

**Table 2:** Average values of common network features for all native models.

Organism	Barycenter	#nodes	#edges	Density	Diameter	Degree	Closeness	Betweenness	Cust. coeff.	Modularity	Communities
*Homo sapiens*^2,50^	HSPA5	216	5286	0.114	7	25	0.0020	145.5	0.522	0.311	5
*Rattus norvegicus*^51,52^	Hspa5	210	3862	0.088	6	18	0.0020	161.2	0.496	0.374	6
*Mus musculus*^53,54^	Hspa5	231	4900	0.092	6	21	0.0018	170.7	0.538	0.354	6
*Macaca fascicularis*^55^	HSPA5	186	2468	0.072	6	13	0.0020	160.5	0.437	0.422	7
*Bos taurus*^56^	HSPA5	205	3292	0.079	8	16	0.0019	172.6	0.447	0.374	6
*Oryctologus cuniculis*^57,58^	HSPA5	174	2598	0.086	6	15	0.0023	134.7	0.464	0.367	5
*Gallus gallus*^59,60^	HSPA5	179	2048	0.064	8	11	0.0020	173.5	0.395	0.385	7
*Danio rerio*^61–63^	hspa5	214	3240	0.071	8	15	0.0018	181.3	0.413	0.375	6
*Drosophila melanogaster*^64–66^	Hsc70-3	57	760	0.238	5	14	0.0089	29.9	0.560	0.324	3
*Caenorhabditis elegans*^67–69^	hsp-90	109	1212	0.103	7	11	0.0034	98.9	0.594	0.450	6
*Saccharomyces cerevisiae*^16,70,71^	KAR2	150	2834	0.127	6	19	0.0028	108.0	0.607	0.352	6
*Arabidopsis thaliana*^72–75^	BIP2; BIP3	62	1188	0.314	5	19	0.0092	25.6	0.680	0.245	3

### Network topology analysis

#### Standard network characteristics

**Barycenter**

We find that the barycenter of all network models corresponds to the Binding Immunoglobulin Protein (KAR2 and BIP2/BIP3 proteins in *Saccharomyces cerevisiae* and *Arabidopsis thaliana* are homologous to vertebrates HSPA5), apart from *Caenorhabditis elegans*, for which the Heat shock protein 90 (hsp-90) results as the key protein (Table 2). Indeed, literature shows that in *Caenorhabditis elegans*, hsp-90 plays a crucial role in the chemotaxis to non-volatile and volatile attractants detected by AWC sensory neurons^76,77^.

**Density**

*Arabidopsis thaliana* and *Drosophila melanogaster* are the most densely connected networks, with values of 0.314 and 0.238, respectively (Table 2). The less densely connected network is found for the invertebrates, except for *Homo sapiens*, with values < 0.100.

**Average degree**

The highest average degree value of the native models is observed for *Homo sapiens* (25) followed by *Mus musculus* (21), *Arabidopsis thaliana*, and *Saccharomyces cerevisiae* with a value of an average degree of 19. The lowest average degree value is found for *Gallus gallus* and *Caenorhabditis elegans*, with a numeric value of 11, followed by *Macaca fascicularis* with a value of 13.

**Closeness centrality**

*Arabidopsis thaliana* has the highest closeness, with a value of 9.2$\cdot$10^-3^, followed by *Drosophila melanogaster* (8.9$\cdot$10^-3^). Vertebrates show similar values, in the range [1.8$\cdot$10^-3^, 2.3$\cdot$10^-3^]. Betweenness centrality. Vertebrates show larger values of betweenness centrality, with the lower end for *Homo sapiens* (145.5) and the upper one for *Danio rerio* (181.3), compared to the remaining organisms.

**Clustering coefficient**

The highest clustering coefficient value is observed for *Arabidopsis thaliana* (0.680) followed by *Saccharomyces cerevisiae* (0.607). All the remaining organisms show a clustering coefficient < 0.600 suggesting that, in the smaller networks, proteins are directly connected with their neighbors.

**Modularity and communities**

**Fig. 2:**
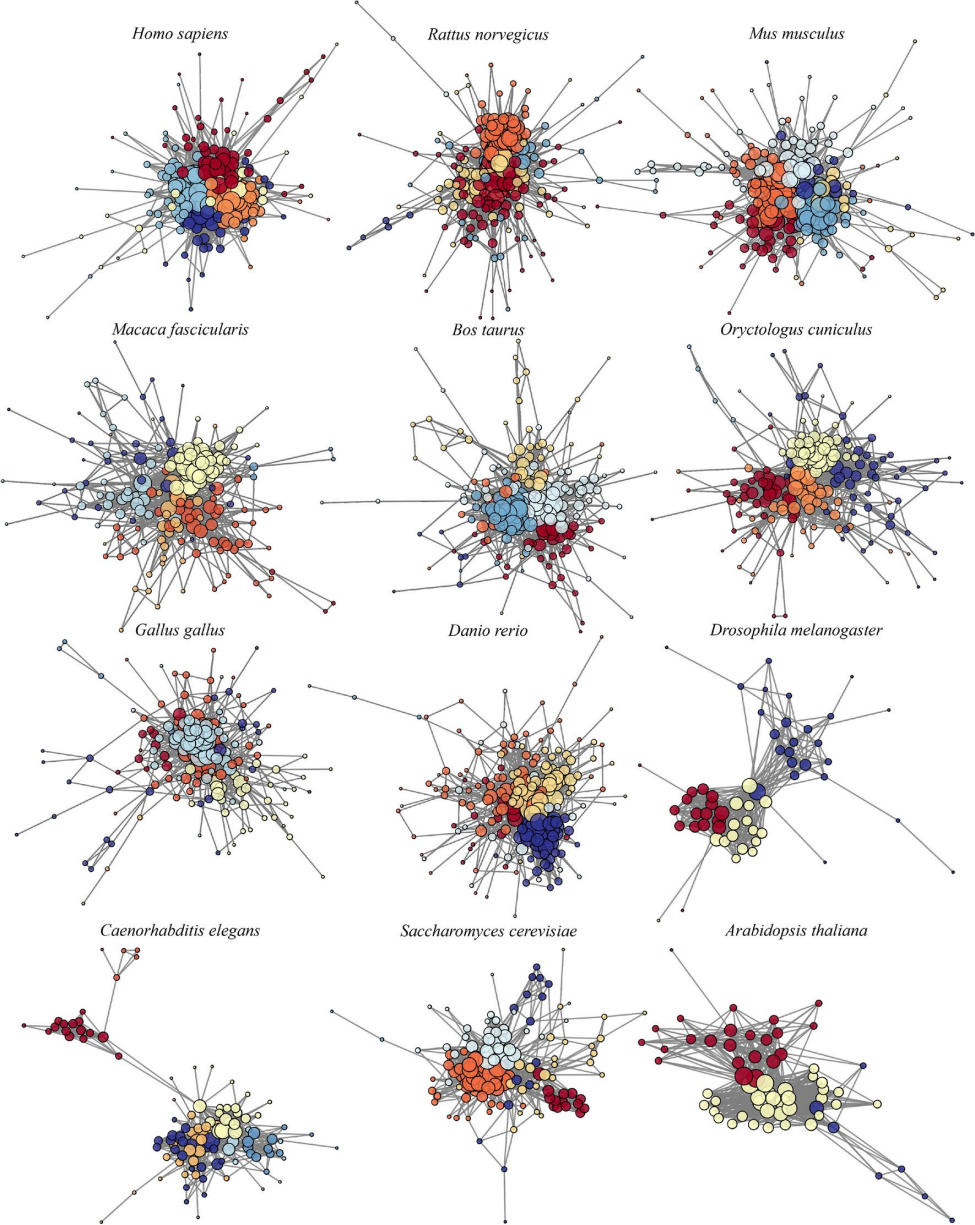
Graph representation of native UPR models across species. Distinct colors identify communities, and the size of the nodes is related to their degree.

The modularity and number of communities provide a different description of alteration in a network since it is an evaluation based on its configuration models. In Table 2 we report the number of communities and values of modularity calculated with the Louvain algorithm. *Drosophila melanogaster* and *Arabidopsis thaliana* provide the same lowest number of communities (3), probably because the sizes of the networks are quite small and comparable among them, concerning the other organisms (57 and 62 nodes). This is also reflected in the smallest diameter of the graph (5 for both). *Rattus norvegicus*, *Bos taurus*, and *Danio rerio* provide the same number of communities (6) and modularity value (0.374/0.374/0.375). A graph representation of the main communities of native models is shown in Fig. 2.

**Correlation analysis**

Pairwise comparisons for degree, closeness, and betweenness are evaluated via the Pearson correlation coefficient. This analysis is performed only across these quantities because they are the more common centrality measures. Degree and closeness centrality show a little negative non-significant correlation (*r* = −0.04, *p* = 0.91), while degree and betweenness centrality show a little positive non-significant correlation (*r* = 0.006, *p* = 0.98). Finally, the two centrality measures show a strong significant negative correlation (*r* = −0.94, *p* = 4$\cdot$10^-6^).

#### Normalized metrics characteristics

The native UPR models for the different organisms are characterized by different sizes that may affect the standard measures reported in the previous paragraph. Thus, when analyzing normalized metrics (based on the network size), analogies and differences between organisms become clearer (Table 3).

**Table 3:** Normalized values and statistical analyses of common network features for native models. p-values are from the non-parametric signed-rank test applied to paired normalized distributions of native and configuration model metrics.

Organism	Degree	Closeness	Closeness	Closeness	Betweenness	Betweenness	Betweenness	Clust. Coeff.
	Norm. value	Norm. value	*z*-score	p-value	Norm. value	*z*-score	p-value	*z*-score
*Homo sapiens*	0.113	0.440	-3.1	$\ll$0.05	0.063	1.9	< 0.05	3.1
*Rattus norvegicus*	0.088	0.408	-3.1	$\ll$0.05	0.042	-1.0	$\ll$0.05	3.1
*Mus musculus*	0.092	0.417	-3.0	$\ll$0.05	0.040	-1.8	$\ll$0.05	3.1
*Macaca fascicularis*	0.071	0.377	-3.1	$\ll$0.05	0.074	-0.3	$\ll$0.05	3.1
*Bos taurus*	0.078	0.386	-3.1	$\ll$0.05	0.053	0.1	$\ll$0.05	3.1
*Oryctologus cuniculis*	0.086	0.404	-3.0	$\ll$0.05	0.065	1.1	$\ll$0.05	3.0
*Gallus gallus*	0.064	0.353	-3.1	$\ll$0.05	0.073	0.6	$\ll$0.05	3.1
*Danio rerio*	0.071	0.383	-3.1	$\ll$0.05	0.072	1.5	$\ll$0.05	3.0
*Drosophila melanogaster*	0.234	0.501	-2.0	< 0.05	0.070	-1.6	> 0.50	3.0
*Caenorhabditis elegans*	0.102	0.368	-3.1	$\ll$0.05	0.048	-2.7	< 0.05	3.1
*Saccharomyces cerevisiae*	0.126	0.422	-3.1	$\ll$0.05	0.092	-1.3	< 0.05	3.1
*Arabidopsis thaliana*	0.309	0.563	-3.1	$\ll$0.05	0.135	0.7	> 0.50	3.0

**Degree**

Except for *Homo sapiens*, the other vertebrates share comparable values of normalized average degree (all with < 0.100). *Drosophila melanogaster* and *Arabidopsis thaliana* have the highest values (> 0.200).

**Closeness centrality**

*Homo sapiens*, *Rattus norvegicus*, *Mus musculus*, and *Saccharomyces cerevisiae* share similar values of normalized average closeness centrality (between 0.404 and 0.440). *Macaca fascicularis*, *Bos taurus*, *Danio rerio*, and *Caenorhabditis elegans* models also provide similar values (between 0.368 and 0.386). *Arabidopsis thaliana* and *Drosophila melanogaster* have the highest closeness values (0.563 and 0.501).

**Betweenness centrality**

Except for *Arabidopsis thaliana* and *Saccharomyces cerevisiae*, which also provide the highest values (0.135 and 0.092), all the other organisms have comparable values of normalized average betweenness centrality, especially the two murine organisms (0.042 and 0.040), and *Macaca fascicularis*, *Gallus gallus*, *Danio rerio* and *Drosophila melanogaster* (0.074/0.073/0.072/0.070)

**Configuration models and** *z*-**score distributions**

We use the configuration models to assess concrete differences between organism models. For each real-world UPR model, we reconstruct 10 configuration models, to compute the *z*-scores of closeness centrality, betweenness centrality, and clustering coefficient, reported in Table 3. *Rattus norvegicus* and *Mus musculus* show comparable values of *z*-scores for all three quantities ([-3.1, -3.1], [-1.8, -1.0], [3.1, 3.1] respectively). The *Drosophila melanogaster* model shows a significant difference from all the other organisms regarding the closeness centrality (the only model with a *z*-score of -2.0). Overall, *z*-scores of closeness and clustering coefficient are included in small variation ranges ([-3.0, -2.0] and [3.0, 3.1] respectively). Moreover, the *z*-score for betweenness centrality shows significant differences among organisms (from -2.7 for *Caenorhabditis elegans* to 1.9 for *Homo sapiens*), suggesting that betweenness centrality provides a potentially useful tool for identifying similarity or differences between organisms regarding this specific mechanism.

**Configuration models and statistical analysis**

Table 3 also shows the nonparametric signed-rank test results for closeness and betweenness centralities. Regarding closeness centrality, we obtain that the native and the configuration models are significantly different for all organisms. All the p-values for closeness are smaller (or much less) than 0.05, so the test rejects the null hypothesis of zero medians at the 5% significance level. On the other hand, for the betweenness centrality distribution, the test provides a non-significant difference for *Drosophila melanogaster* and *Arabidopsis thaliana*. Overall, the configuration models alter the network features regarding closeness and only partially regarding the betweenness centrality.

**Highest metrics nodes**

**Fig. 3:**
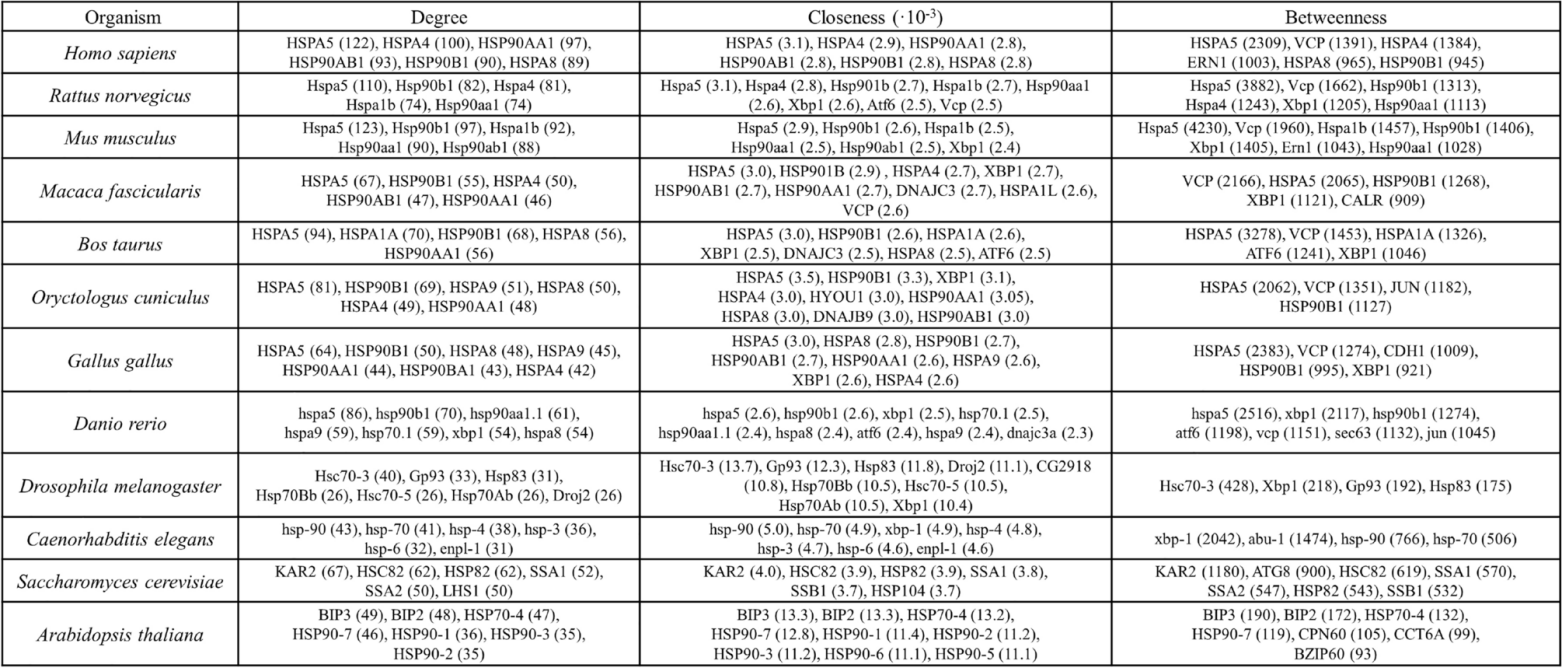
Protein sets with highest values of network metrics, for native models. Values are reported in round brackets after the protein name.

To more precisely relate the evaluated network metrics with the biological content, we also analyze the role of protein within the UPR pathways. Few specific genes or their homologues appear in all the sets across organisms, associated with high degree, closeness, and betweenness centralities. In Fig. 3 we show the nodes with the highest values of the three principal metrics, with the values in the round brackets.

A percentage varying between 5% and 30% of the total number of proteins in each native network is represented by heat shock cognate proteins (HSC), which are members of the heat shock protein family (HSP), one of the most ubiquitous and conserved protein families across organisms^78–81^. They are fundamental in the correct functioning of cells, maintaining cellular proteostasis, and protecting cells from induced stresses^82^. Their genes are associated with the highest values of the main three network features, across all the twelve species. They result in relevant nodes and hubs in various diseases, like cancers and strokes^83–85^. HSP/HSC proteins for each organism belong to the same module, associated with the largest node size (Fig. 2). Other proteins related to the highest values of the three metrics for the various networks are i) X-box binding protein 1 (XBP1), which is an important initiator and modulator factor of ER stress response^86,87^, ii) ATF6 and iii) ERN1, both of which are ER membrane receptors (together with EIF2AK3)^9,88–90^, in charge of initiating and regulating the stress response after the activation promoted by HSPA5/BiP^91–93^. The most relevant pathway in the UPR mechanism for *Saccharomyces cerevisiae* and *Arabidopsis thaliana*is related to the IRE1/ERN1 signaling cascade^94–99^, also notable from the fact that we cannot find homologs for the other two ER stress sensors. Finally, a recurrent protein among vertebrates is the Valosin-containing protein (VCP) which is one of the most abundant cytoplasmic proteins in eukaryotic cells^100^; its main function is to mediate protein quality control processes to maintain cell homeostasis, like ERAD^101^.

**Fig. 4:**
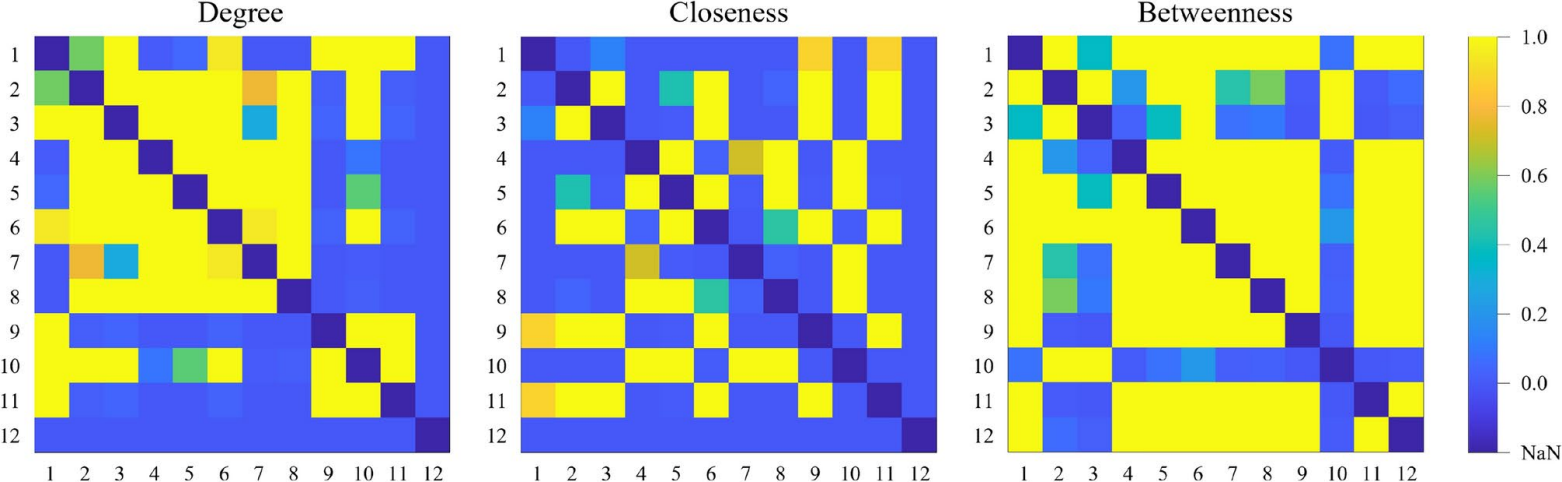
Correlation matrices (p-value) of normalized metrics distributions. Matrix indices represent the organisms as listed in the text. The color scale is the same for all the matrices (color bar on the right). NaN elements identify the diagonal of the matrices (same organism).

**Multi-comparison test** In Fig. 4 we report the correlation matrices related to multiple comparison tests with the Bonferroni correction applied to normalized metrics distributions (degree, closeness centrality, and betweenness centrality) cross-species. The representation of metrics distributions is shown in Fig. S5 of Supplementary material.

Degree − We observe that normalized degree strong similarities among vertebrates (big yellow square between 2−*Mus musculus* and 8−*Danio rerio*). Interestingly, *Homo sapiens* distribution is similar, besides the two murine models and the rabbit, to *Drosophila melanogaster*, *Caenorhabditis elegans*, and *Saccharomyces cerevisiae*. Nicely, *Caenorhabditis elegans* distribution is similar to 7 out of 12 organism models.

Closeness centrality − This feature distributions show a strong overlap between *Rattus norvegicus* and *Mus musculus*. Interestingly, these two models together with the human and the rabbit produce a similarity with *Drosophila melanogaster* and *Saccharomyces cerevisiae*, while *Macaca fascicularis*, *Bos taurus* *Gallus gallus* and *Danio rerio* models produce a similarity with *Caenorhabditis elegans*.

Betweenness centrality − On the contrary, the correlation matrix of betweenness centrality shows that most organisms have identical normalized distributions (most matrix elements are closer to 1), so this metric does not discriminate among all organisms.

We can conclude that statistical and correlation analyses show that degree and closeness centrality result in better network features to discriminate across organisms. Fig. S6 of Supplementary material shows the dendrograms of hierarchical analysis of normalized average degree and centralities. The degree and closeness trees identify similarities among vertebrates, and nicely with *Caenorhabditis elegans* and *Saccharomyces cerevisiae* (animal models). In all cases, the two murine models are among the most similar. Fig. S7 of Supplementary material shows the dendrograms of hierarchical analysis of normalized median of degree and centralities distributions (Fig. S5). The situation slightly changes from the analysis on average values. Again, *Rattus norvegicus* and *Mus musculus* are grouped as similar. Nevertheless, closeness centrality confirms the phylogenetic hierarchy obtained with average values; the degree groups the murine models with fruit fly and worm models, while *Arabidopsis thaliana* is grouped with the rest of the invertebrates. Betweenness centrality now provides a situation analog to the hierarchical organization of the degree for average values.

**Topological analysis**

A different type of information comes from the topological analysis of adjacency matrices, evaluated with the Generalized Hamming Distance (GHD, Table 4). It provides a degree of difference between two $N \times N$ matrices, by comparing paired matrix elements^49^. In Table 4 we report the average value of GHD calculated for the native network concerning all the 10 associated configuration models. The most different models are provided by *Drosophila melanogaster* (0.219), *Arabidopsis thaliana* (0.198), and *Caenorhabditis elegans* (0.135); this can be rationalized because the size of the network is small compared to the other organisms, and we have poor biological information about the nodes, so the null models generate quite different related networks. All vertebrate models provide small and comparable GHD values (< 0.100), especially *Mus musculus* (0.051), *Homo sapiens* (0.057), *Danio rerio* (0.059), and *Rattus norvegicus* (0.060).

**Table 4:** Average Generalized Hamming Distance between native and configuration models.

*H. sapiens*	*R. norvegicus*	*M. musculus*	*M. fascicularis*	*B. taurus*	*O. cuniculis*	*G. gallus*	*D. rerio*	*D. melanogaster*	*C. elegans*	*S. cerevisiae*	*A. thaliana*
0.057	0.060	0.051	0.076	0.064	0.076	0.071	0.059	0.219	0.135	0.095	0.198

To sum up, degree and closeness centrality are convenient network features since they discriminate across different organisms and identify native or null networks within the same organism. Summarizing the results for all the above quantities, the vertebrates (*Homo sapiens*, *Rattus norvegicus*, *Mus musculus*, and *Bos taurus*) and their biological models (*Danio rerio*, *Caenorhabditis elegans*, and *Saccharomyces cerevisiae*) share similar values, in particular of closeness, and have similar connections, as shown by the correlation matrices and the GHD values. Overall, the description of UPR networks via standard network quantities and GHD allows us to identify phylogenetic similarities and characterize the networks of vertebrates concerning other phyla.

### Network robustness

**Fig. 5:**
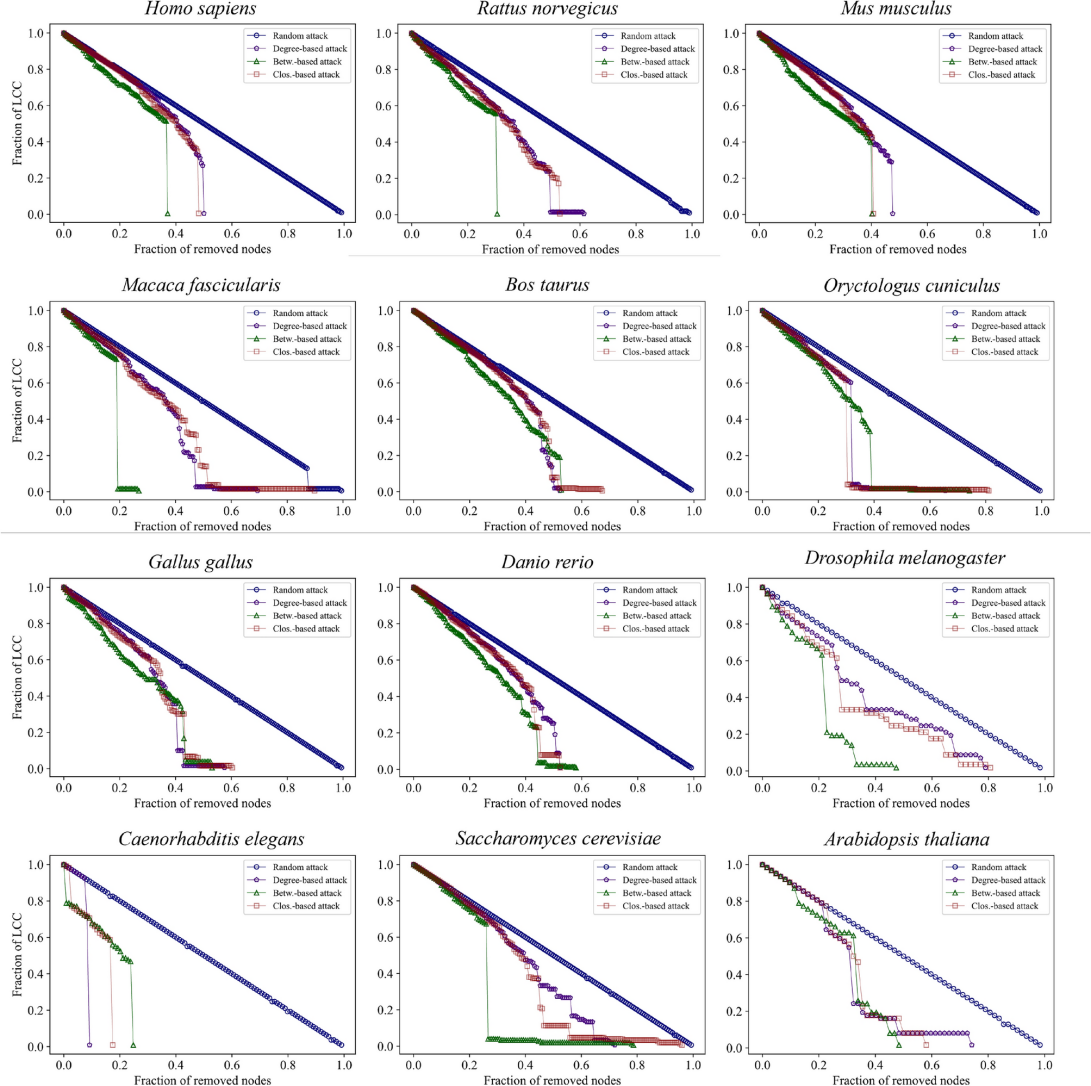
Network robustness evaluated using random attack and target attacks on native models. Different colors identify attack strategy trends.

The network robustness (Fig. 5) has been tested with random- and metrics-based target attacks on the native networks. Table 5 reports normalized average path length and efficiency values for all investigated models. The average shortest path length values are comparable across organisms (from the lowest value for *Drosophila melanogaster* (2.068) to the largest value for *Gallus gallus* (2.949)). Murine models have the highest efficiency values (> 0.600), which also share comparable values of average path length.

**Table 5:** Values of average path length and efficiency of all native models.

Organism	Average path length	Efficiency
*Homo sapiens*	2.354	0.488
*Rattus norvegicus*	2.543	0.638
*Mus musculus*	2.485	0.681
*Macaca fascicularis*	2.735	0.446
*Bos taurus*	2.693	0.586
*Oryctologus cuniculis*	2.557	0.593
*Gallus gallus*	2.949	0.504
*Danio rerio*	2.702	0.551
*Drosophila melanogaster*	2.068	0.571
*Caenorhabditis elegans*	2.832	0.429
*Saccharomyces cerevisiae*	2.450	0.478

The evolution of the largest connected component (LCC) in each network is evaluated by removing at each step one node from the network based on ascending index (random)^102^, degree^103^, and centralities^104^ attacks, i.e. the least “important” nodes are removed first. The LCC identifies a connected component of a given graph that contains a significant fraction of the entire graph’s vertices. The removal based on random node choice provides a linear trend for all organisms (Fig. 5). A random attack strategy (based on random node removal) requires the removal of many nodes to decrease the potency significantly, so targeted attacks are more efficient in degrading the network^105,106^. In most cases, the betweenness-based attack strongly affects the behavior of the network, except for *Caenorhabditis elegans*, for which the opposite is observed. In general, degree-based and closeness-based attacks show the same behavior of LCC degradation. This finding is also supported by sets of nodes with the highest values of metrics (see Fig. 3), where it is shown that sets for degree and closeness are more similar compared to sets obtained for betweenness centrality, as also confirmed by the computed correlation coefficients between network metrics (see above). Furthermore, vertebrate models show similar behaviors to all attacks.

By removing nodes with increasing metrics values, we expect a slower degradation of the network integrity; the results could be explained by considering a larger number of “external” and less important nodes within the networks (low values of network metrics). Attacking the networks based on decreasing metrics values provides a random-like trend in network robustness. The sudden jumps in the robustness can be associated not only with a precise role of a specific protein removal, because their removal at the beginning of the process could not produce the same results, as in a purely star network. Instead, it can also be the result of a cumulative effect, because the overall removal of all the previous proteins builds a star-like network, that collapses upon removal of a specific protein.

However, going through the principal functions and pathways that are involved in the UPR and arising from the network robustness, across all, or most of, the organisms, we highlight that those proteins with the role of chaperones, i.e. initiating the signaling pathways and quality control of protein folding, are present in all considered organisms (except *Homo sapiens*) as the ones that induce a sudden collapse of metrics robustness. Also, proteins involved in pro-apoptosis or ERAD mechanisms emerge from the robustness analysis as present in most organisms. Other mechanisms, with corresponding proteins obtained from robustness stress analysis, are listed in Table 6.

**Table 6:** Proteins and functions related to jumps in the robustness trends. MP,R,&T = membrane proteins, receptors, and transfers; R&M = regulators and messengers; I&T factor = initiation and transcription factors. Autoph./Aptopt. = autophagy/apoptosis. Jump-related proteins are considered when the LCC dimension decreases by at least 10%.

Organism	Chaperone	MP,R,&T	Kinase	R&M	ERAD/Autoph./Pro-survival	Apopt.	I&T factors	Protein folding
*Homo sapiens*	−	−	MAPK8	MAPK8	TMBIM6, THBS4	MAPK8	−	−
*Rattus norvegicus*	CCt5, Dnajb2	Ep300, Dnajb2	−	Dnajb2	Dnajb2	−	−	CCt5
*Mus musculus*	Bag3	−	−	−	Bag3, Creb3, Manf	−	Creb3	−
*Macaca fascicularis*	HSPA9	−	−	DDIT3	−	HSPA9, DDIT3,BCL2L11	DDIT3	HSPA9
*Bos taurus*	DNAJA1, HSPA4L	−	−	HSF1	−	DNAJA1, HSF1	HSF1	−
*Oryctologus cuniculis*	DNAJC10	VAPB, ERO1B	−	−	DNAJC10, VAPB	DNAJC10	−	ERO1B
*Gallus gallus*	HSPD1, HSPA4, PDIA6	−	−	HSPA4	PDIA6	−	−	HSPD1, ERP44, HSPA4, PDIA6
*Danio rerio*	bag6, hyou1, cryaa	−	−	hyou1	bag6, hyou1, cryaa	bag6	−	hyou1, cryaa
*Drosophila melanogaster*	Gp93, DnaJ-1, Calr, Grp170	Calr	−	Dad1	Dad1, Der-2	−	−	Gp93, DnaJ-1, Calr, Grp170
*Caenorhabditis elegans*	enpl-1	col-109, abu-6, F38B6.6, abu-12	−	−	col-109, abu-6, enpl-1, abu-12	F38B6.6	pqn-90, enpl-1	−
*Saccharomyces cerevisiae*	CCT3, HSP10	BUD27	−	−	EDE1	−	−	BUD27, CCT3, HSP10
*Arabidopsis thaliana*	HSP90-3, HSP90-3, BIP3, HSP70-7, HSP70-10	−	−	−	HSP70-7, HSP70-10, BIP3	−	−	HSP70-7, HSP70-10, BIP3

We report the network robustness analysis applied to the configuration models in Fig. S8 of Supplementary material. We choose the nearest and the farthest models from the native models based on the network topology (lowest and biggest dGHD values) to investigate the behavior concerning the randomized networks. The nearest topology network of *Homo sapiens*, *Rattus norvegicus*, *Mus musculus*, *Gallus gallus*, and *Saccharomyces cerevisiae* shows a stronger resistance to fails than the farthest topology network (similar to the native models − Fig. 5). In addition, there is a (nearly) complete overlap of the three targeted attack trends for all organisms.

### Structural controllability

The structural controllability allows for determining the number and identifying the minimum driver nodes (Table 7). For all organisms, the study of the native model may produce different driver node sets; however, some proteins recur in sets across organisms, suggesting their importance within the networks and in the mechanism. Unfortunately, the algorithm cannot find any driver node set for the bull model, as it is.

**Table 7:** Structural controllability results for all species models (DNs (set dimension) and number of possible node sets). Identified proteins for each model are listed in the round brackets.

Organism	Driver nodes	# possible sets
*Homo sapiens*	1 (RPAP2)	1
*Rattus norvegicus*	3 (Ufl1, Rhbdd2, Krt14/Krt16)	2
*Mus musculus*	4 (Ficd, Parp8, Crebzf/Crebrf, Il24/Igtp)	4
*Macaca fascicularis*	4 (TOR1B, SIAH2, HSP1/HSPA4L, UFL1/DDRGK1)	4
*Bos taurus*	−	−
*Oryctologus cuniculis*	3 (FAP/LOXL2, FICD/SERPINH1, PTPN2/TNIK)	8
*Gallus gallus*	3 (KRT6A, KRT4/KRT14, CDKSRAP3/UFL1)	4
*Danio rerio*	2 (tnika, parp6b/parp8)	2
*Drosophila melanogaster*	2 (Hsp70Ab, L(2)efl)	1
Caenorhabditis elegant	3 (abu-12, abu-5/pqn-79, pqn-70/pqn-90)	3
*Saccharomyces cerevisiae*	4 (TSR4/SYO1, NUP42/ASM4, NUP1/NUP60, NUP145/NUP159)	16
*Arabidopsis thaliana*	9 (DJA6, GFA2, T14C9.70/T13C7.14/T17F15.190/F14N23.23/F23H11.4/T10O8.100/T16O11.15/DJA5)	8

The algorithm finds a unique set only for *Homo sapiens* and *Drosophila melanogaster*models. RPAP2 is associated with transcription and RNA processing, connects to RUBVL2, and has minimal network metrics^107^. In metrics terms, this node is characterized by 1 degree (connected to RUBVL2), 1.48$\cdot$10^-3^closeness centrality, and 0 betweenness centrality (we can define it as an “external node”). In fruit flies, Hsp70Ab, which belongs to the Hsp70 family, stabilizes non-native protein conformations^108,109^. The L(2)efl protein^110^ induces phosphorylation of Eif2$\alpha$, playing a role in aging^111–113^.

The FIC domain protein adenylyltransferase (Ficd) is found in *Mus musculus* and *Oryctologus cuniculis*. Ficd is associated with various cellular pathways, particularly the ATF6 and EIF2AK3 branches of the UPR pathway, which regulate ER homeostasis. In humans, FICD is typically present at low basal levels in most cell types, and its expression is tightly regulated^114,115^.

Nicely, the Ufm1-specific ligase 1 (Ufl1)^116,117^ is found in *Rattus norvegicus*, *Macaca fascicularis*, and *Gallus gallus*. The Ufl1 protein is essential for activating the stress sensor EIF2AK3 and helps protect cardiomyocytes from cell death triggered by ER stress.

Kinesins (Krt4, Krt14, and Krt16) are found in *Rattus norvegicus* and *Gallus gallus*^118,119^.

The gene family activated in blocked UPR (abu) in *Caenorhabditis elegans*is implicated in the UPR regulation in response to the ER stress. They help maintain protein-folding homeostasis and manage the accumulation of misfolded proteins in the ER^69^. This class is a subset of the pqn family, prion-like Q/N proteins^120^.

Interestingly, many resulting proteins are chaperones, such as heat shock proteins (HSP) and DnaJ homologues (i.e., all *Arabidopsis thaliana* proteins). Their chaperone and protein-folding regulation activity is well known.

## Discussion

Unfolded Protein Response is one of the most conserved fundamental biological mechanisms through organisms^15,16,75,121^, occurring primarily in the endoplasmic reticulum (ER). Its main function is to restore cell homeostasis after a pathological accumulation of non-properly folded proteins^6,7,10,11,122,123^. Any inefficiency in the adaptive response to ER stress can lead to unfolded or misfolded protein accumulation at different levels. These proteins tend to aggregate, posing a threat to cellular and tissue integrity and serving as a primary driver for the onset of amyloidosis and neurodegenerative diseases^25–27,124^.

A key signaling pathway governing the UPR, originally discovered in *Saccharomyces cerevisiae*during the 1970s^16,50,125,126^, is characterized by a single transmembrane protein, ERN1, responsible for the detection of ER stress provoked by over-accumulation of unfolded/misfolded proteins^94–96^. The major ER chaperone BiP triggers the dimerization of ERN1, which leads to its subsequent autophosphorylation and the activation of its signaling cascade^9^. This pathway reinforces the ER function and is conserved across eukaryotes^127^. Indeed, the basic features of the UPR mechanism result in being highly preserved throughout metazoans; most species have homologues of the three main stress sensors, ERN1, ATF6, and EIF2AK3. Signaling pathways of the stress sensors cooperate to restore and/or bolster ER function, primarily through the upregulation of many components of the protein folding machinery (as the action of XBP1 on the regulation of BiP chaperone^86,87^within the ERN1 pathway) and the quality control machinery within the ER. Additionally, these signaling pathways limit ER stress by dampening the translation attenuation and potentially engaging the regulated ERN1-dependent decay (known as RIDD)^128–130^. A network-based description of cell mechanisms, using protein-protein interactions (PPI) networks, offers a valuable tool for comprehending the behavior of complex systems. Some points must be considered before discussing the analysis results. PPI network models are built utilizing biological data from publicly available PPI databases. These databases collect various types of data, from thousands of experimental works. Despite their large size, the databases are not exhaustive, because only part of the molecular pathways have been completely understood and characterized. The absence or bias in information stored in interaction databases must be considered when creating and analyzing biological networks. PPI databases must contain sufficient information for a specific pathway to yield qualitative accurate results from network analysis techniques.

With these premises, we apply a classical network metrics investigation to assess the similarities and differences among UPR models from twelve different organisms, considering the existing phylogenetic pathway in all the analyzed models. As model organisms, we choose for our investigation *Homo sapiens*, *Rattus norvegicus*, and *Mus musculus*due to the quite complete genomic accordance with human^131–136^ and other model organisms, as *Macaca fascicularis*^55^, *Bos taurus*^56^, *Oryctologus cuniculis*^57,58^, *Gallus gallus*^59–63^, *Drosophila melanogaster*^64–66^, *Caenorhabditis elegans*^67–69^, *Saccharomyces cerevisiae*^16,70,71^, and *Arabidopsis thaliana*^72–75^.

Comparisons made for common metrics provide that network measures are sensitive to the network features (Table 2, and Figs. S2, S3, S4 from Supplementary material); however, from a biological point of view, the network analysis shows that there are strong similarities among the model organisms (Table 3), in particular among vertebrates. Moreover, highly conserved genes and pathways across species arise (Fig. 3). Chaperones belonging to the heat shock protein (HSP) family are among the most central proteins regarding network metrics, as the densest connected, and they also play a fundamental role in the correct functioning of cells^78–82^. This result can be interpreted as support for the significance of these proteins, as it implies a correlation between the structure and the biological properties of protein networks. In summary, network theory and statistical mechanics confirm that it is possible to identify similarities among organisms phylogenetically related via their PPI networks. In addition, we find that some network metrics are better at discriminating among organisms; they are useful for identifying similarities and differences, such as closeness centrality and node degree.

Additionally, in this study, we employ a network scientific methodology to investigate the tolerance levels of multiple systems when subjected to external perturbations. We achieve this by adopting a measure of network robustness^137–140^, to characterize the potential resilience of several PPI networks. The robustness of the twelve organism models is evaluated by removing nodes, and consequently altering the network integrity, adopting different network-based metrics target strategies (Fig. 5). Obviously, in these cases, network robustness is influenced by network features and the degree of accuracy of biological information accessible online, but similarities in resilience behavior arise for organisms that are phylogenetically closer, for example, *Homo sapiens*and murine species, despite the difference between network features. This result can be supported by the closeness between these organisms in terms of phylogeny since 99% of the genome is conserved between human and murine species^131–136^. A detailed analysis of genes related to jumps in the network robustness highlights that, despite different proteins being involved in the organisms, there is a recurrence of pathways across most species. In particular, the sensing role of the chaperones is fundamental, together with the apoptosis and endoplasmic-reticulum-associated protein degradation (ERAD) functions (Table 6). Lastly, the minimum drive nodes methodology, based on the structural controllability analysis^41,42^and employing Kalman’s rank condition^141^, yields significant biological insights into the proteins involved in specific mechanisms. It becomes feasible to pinpoint the key nodes that exert control over the entire network^142^. We apply structural controllability to all organism models investigated in this work. Driver node sets are related to the topological structure of the network; as for the other network methods applied, results are sensitive to the biological information available online (also in the high variability of the possible DNs sets − several sets for the same organism in Table 7). Attributing a biological significance to the set of driver nodes is not straightforward. When analyzing networks with numerous nodes and edges, the possibility of losing biological information in the models is due to the consequent increase of missing information stored in the databases. However, some specific mechanisms and genes, already identified via robustness and network statistics, also arise through structural controllability.

This study represents a comprehensive and innovative analysis of the biological behavior and characteristics of a fundamental cellular control mechanism, the Unfolded Protein Response (UPR). By modeling the UPR as a protein-protein interaction (PPI) network, it uses standard and advanced techniques to extract meaningful information from the network. The study establishes a direct correlation between specific network features and biological components. It finds that the three methods employed − standard network metrics and topology, network robustness, and network control theory − offer complementary and non-conflicting characterizations of the systems studied.

The first class of methods offers a comprehensive portrayal of the PPI networks and discerns phylogenetic similarities, distinguishing vertebrates from organisms of other phyla. Additionally, it identifies key genes (network nodes) that significantly influence the overall UPR mechanism. Network robustness utilizes metrics to assess the network’s ability to withstand node removal, identify pivotal proteins and sub-pathways, and simulate disease onset. This robustness analysis also highlights phylogenetic similarities among organisms.

Among the various UPR sub-mechanisms, chaperone activity, apoptosis, and ERAD are identified as relevant. Though less distinctly, control theory also pinpoints proteins with central roles, particularly chaperones, transcription factors, and ERAD proteins. Using network models for molecular and cellular pathways is a powerful yet underutilized approach. This study’s combination of diverse network descriptors and methods provides profound insights into complex mechanisms. It emphasizes revising and enriching PPI databases to create increasingly accurate biological models.

## Materials and methods

Here we present a study of the unfolded protein response mechanism among twelve organisms. Below we report general information of the network models investigated in this study and the description of the performed analyses. We perform calculations of i) network descriptors, ii) modularity and communities, iii) topological distance, iv) network robustness, and v) structural controllability. We perform comparisons among native models, with the help of the configuration models (degree-based reconstruction). Native models are built using Python v. 3.11 programming language and NetworkX Python library^143^, based on information on the paired connection between couples of nodes, and configuration models are built using MATLAB v. R2023a programming language. Modularity, community detection, and robustness analyses are performed using the NetworkX library, while network descriptors analysis and structural controllability^144,145^ have been implemented in MATLAB.

### Unfolded protein response network models

Here we present the UPR network models proposed in this work. Investigated organisms are *Homo sapiens*, *Rattus norvegicus*, *Mus musculus*, *Macaca fascicularis*, *Bos taurus*, *Oryctologus cuniculis*, *Gallus gallus*, *Danio rerio*, *Drosophila melanogaster*, *Caenorhabditis elegans*, *Saccharomyces cerevisiae*, and *Arabidopsis thaliana*. We combine information stored in the UniProt database^146–148^and the String protein-protein interaction database^149,150^ to identify proteins involved in the mechanism and build undirected network models. Since the available information in the UniProt database is poor for the vertebrates (old network features of organisms models are reported in Table S1 of Supplementary material), we start from the proteins found for *Homo sapiens*, which results as the most characterized organism, to reconstruct the PPI networks for *Rattus norvegicus*, *Mus musculus*, *Macaca fascicularis*, *Bos taurus*, *Oryctologus cuniculis*, *Gallus gallus*, and *Danio rerio*. We therefore combine the proteins listed in the UniProt database for the specific organism with the human list. In addition, we manually curate the literature to include the proteins (chaperone BiP and three stress sensors with their pathways) from previous work^151^ in the vertebrate models. We impose a minimum required interaction score of medium confidence (0.400, i.e., useful interactions to build the networks must have scores $\ge$ 0.400), and we construct the set of connections among nodes considering experimental evidence, curated databases, text mining, and co-expression associations. To analyze the networks, we download the lists of paired interactions as Tab Separated Values (TSV) extension files from the STRING website. In this framework, original models, also named native, are built directly from biological information obtained from the databases, and connections in configuration models are semi-randomly built based on the total degree of each node in the original models. The matrix representation of native UPR models is shown in Fig. S1 of Supplementary material.

### Analysis of network models using network metrics

We first characterize the models using the usual metrics of network descriptors, (i) to highlight differences and similarities between models related to different organisms and (ii) to evaluate the influence of constructing a network with random connections starting from the degree of nodes of original models. For each model, we calculate (i) the total degree of the nodes, (ii) the betweenness and closeness centralities, which are measures of how often each graph node appears on the shortest path between two nodes in the graph, (iii) local clustering coefficient, and (iv) the modularity and number of communities, as measures of the structure of networks to evaluate the strength of division into different modules, calculated using the Louvain community detection Algorithm. As a definition, networks with high modularity have dense connections within communities. In addition, we also include normalized values of degree (norm(deg) $= 2M/N$, where 2*M* is the total degree of a node in a *N*nodes network)^152^, betweenness (norm(bet) = (bet−min(bet)/(max(bet)−min(bet))) and closeness centrality (norm(clos) = $(N-1)$ $\cdot$clos)^153^ (Table 3). All measures are normalized by removing the dependence from the network dimensionality (normalized distributions shown in Figs. S2, S3, S4 of Supplementary material). We compute z-scores of averaged normalized metrics distributions, defined as $(x-\mu )/\sigma$, where *x* is the variable value, $\mu$ and $\sigma$ are the population’s mean value and standard deviation, respectively. Highest metrics (degree and centralities) proteins are extracted directly from metrics distributions.

### Topological differences between native and configuration models

As well explained in our previous work^151^, we use the algorithm relies on the Generalized Hamming Distance (GHD)^49^, which can be used for assigning a “weight” to the topological difference between networks and evaluating its statistical significance, based on comparison between matrix elements. We apply this theory to assess the difference between the original models (built from the databases) and the configuration models (re-created from the original models). If we consider two distinct networks, labeled *X* and *Y*, with the same number of nodes (*N*), we can calculate the distance dGHD between the two networks as follows:

1 $$\begin{aligned} \textrm{dGHD}(X,Y)= \frac{1}{N \cdot (N-1)} \sum _{i\ne j} (x'_{ij} - y'_{ij}) \end{aligned}$$

where $x'_{ij}$ and $y'_{ij}$ are mean-centered edge-weights, and depend on the network topology, providing a measure of connectivity between every pair of i^th^ and j^th^ nodes in *X* and *Y*, respectively.

### Statistical analysis of network models

The graph analysis and network robustness are performed using Python 3.11^154^. The statistical analysis, the implementation of structural controllability, and the GHD algorithm are performed using MATLAB R2023a^155^. We apply non-parametric tests because our variables are not normally distributed. For paired comparisons between the centralities distributions of native and null models considering one specific organism we use the Wilcoxon signed-rank test, and for non-paired comparisons across different organisms we use a multi-comparison test computing values with the Bonferroni method^156,157^, on the results of a one-way Kruskal Wallis analysis of variance (shown in Fig. S5 of Supplementary material). If *p* < 0.05, the results are considered as statistically significant.

### Network robustness exploitation

Considering a network *X*, composed of *N* nodes set denoted as $V = \{v_1, v_2, \ldots , v_N \}$, interconnected by *M* links represented by $E = \{ (v_i, v_j): v_i, v_j \in V \}$, the robustness *R* of the *X*network is defined by the ratio^43,158^

2 $$\begin{aligned} R = \frac{1}{N} \sum _{i=1}^N G_i \end{aligned}$$

where $G_i=n_i/N$ is the size of the large connected component after the i^th^ node removal; normalization factor $N^{-1}$ is useful for comparing networks of different sizes. Some metrics can be used to quantify a network’s robustness. The average path length *l*can provide a quantification of network robustness since large values mean that nodes are farther apart from each other, and the removal of a node can significantly increase the average paths between nodes, decreasing the robustness of the network^159^:

3 $$\begin{aligned} l = \frac{1}{N \cdot (N-1)} \sum _{i \ne j} d_{ij} \end{aligned}$$

where the sum of all possible paired-node distances is normalized over all the possible couples of *N* nodes. Another useful metric to quantify network robustness is the variation of the efficiency $\Delta E$depending on an increasing number of removed nodes^43^. A robust network would have a small drop in network efficiency with node removal.

4 $$\begin{aligned} \Delta E_i = \frac{E-E_i}{E} \end{aligned}$$

where the efficiency is defined as $E = (N \cdot (N-1))^{-1} \sum _{i \ne j} d_{ij}^{-1}$. In the investigation presented here, the theory of network robustness is employed to assess the capability of networks to deliver and maintain an acceptable level of service in the presence of faults, as outlined by our models. Our analysis involves subjecting each network to random and targeted attacks, following specific strategies (degree-, closeness-, and betweenness-based).

### Structural controllability and minimum driver node identification

Lastly, we employ the structural controllability theory to assess the nodes’ ranking using Kalman’s rank condition for continuous linear time-invariant systems^141^. In addition, we also implement the Minimum Driver Nodes (MDN) algorithm^144,145^, proposed by Liu et al., which is based on the minimal set of input signals required to control the network, and the MDN selection algorithm used by Zhang et al^142^., which can be used to identify the driver nodes − the nodes on which an input signal must be injected to obtain full control of the network. Generally, the time-evolution of a network system consisting of *N* nodes and *M* input signals, with $M \le N$, can be described with the following linear differential equation

5 $$\begin{aligned} \frac{d \textbf{x}(t)}{dt} = \textbf{Ax}(t) + \textbf{Bu}(t) \end{aligned}$$

where $\textbf{x} = (x_1, x_2, x_3, \ldots , x_N)^{\textrm{T}}$ is the state vector for the N nodes system and $\textbf{u} = (u_1, u_2, u_3,\ldots , u_M)^{\textrm{T}}$ is the control vector. **A** is the $N \times N$ state matrix, in which each element $a_{ij}$ identifies the connection between the i^th^ and j^th^ nodes. **B** is the $M \times N$ control matrix, whose dimension *M* depends on the number of input signals:

6 $$\begin{aligned} \textbf{B} = (e_1^{\textrm{T}}~e_2^{\textrm{T}}~e_3^{\textrm{T}}~\ldots ~e_M^{\textrm{T}}) \end{aligned}$$

where $\{ e_1, e_2,e_3,..., e_M \}$ are the vectors of the canonical base. Given **A** and **B**, it is possible to assemble the controllability matrix **C**:

7 $$\begin{aligned} \textbf{C} = (\textbf{B},~\textbf{AB},~\textbf{A}^2 \textbf{B},~\textbf{A}^3 \textbf{B},~\ldots ,~ \textbf{A}^{N-1} \textbf{B}) \end{aligned}$$

The network is fully controllable if the controllability matrix has a full rank, i.e., $\textrm{rank}(\textbf{C}) = N$. Theory and algorithms are well-explained in Ref^151^.

## Supplementary Information

Supplementary Information.

## Data Availability

The datasets generated during and analyzed during the current study are available from the corresponding author upon reasonable request. Source code (Python and MATLAB scripts) used during the current study are available at https://github.com/NLuchetti/StatMech_of_UPR.git.
