# Supplementary material for: A statistical mechanics investigation of unfolded protein response across organisms
Source: Sci Rep. 2024 Nov 12;14:27658. doi: 10.1038/s41598-024-79086-8 (PMC11557608; doi:10.1038/s41598-024-79086-8)
Supplement: Supplementary file 1 — Supplementary Information. [file 41598_2024_79086_MOESM1_ESM.docx]

# Supplementary material of “A statistical mechanics investigation of Unfolded Protein Response across organisms”

Nicole Luchetti^1,2,*^, Keith M. Smith^3^, Margherita A. G. Matarrese^1^, Alessandro Loppini^4^, Simonetta Filippi^1,5,6,*^, Letizia Chiodo^1^

^1^ Department of Engineering, Università Campus Bio-Medico di Roma, Via Alvaro del Portillo 21, 00128 Rome, Italy

^2^ Center for Life Nano- & Neuro-Science, Istituto Italiano di Tecnologia, Viale Regina Elena 291, 00161 Rome, Italy

^3^ Computer and Information Sciences, University of Strathclyde, 26

Richmond Street, Glasgow, G1 1XH, United Kingdom

^4^ Department of Medicine and Surgery, Università Campus Bio-Medico di Roma, Via Alvaro del Portillo 21, 00128 Rome, Italy

^5^ National Institute of Optics, National Research Council, Largo Enrico Fermi 6, 50125 Florence, Italy

^6^ International Center for Relativistic Astrophysics Network, Piazza della Repubblica 10, 65122 Pescara, Italy

Table S1: Average values of common network features for original native models.

|  | Barycenter | #nodes | #edges | Density | Diameter | Degree | Closeness | Betweenness | Cust. coeff. | Modularity | Communities |
| --- | --- | --- | --- | --- | --- | --- | --- | --- | --- | --- | --- |
| *Homo sapiens* | HSPA5 | 216 | 5286 | 0.114 | 7 | 25 | 0.0020 | 145.5 | 0.522 | 0.311 | 5 |
| *Rattus norvegicus* | Hspa5 | 139 | 2224 | 0.116 | 5 | 16 | 0.0030 | 95.3 | 0.533 | 0.368 | 5 |
| *Mus musculus* | Hspa5 | 172 | 3200 | 0.109 | 6 | 19 | 0.0025 | 119.1 | 0.548 | 0.352 | 6 |
| *Macaca fascicularis* | HSPA5 | 64 | 578 | 0.143 | 6 | 9 | 0.0067 | 45.8 | 0.544 | 0.419 | 5 |
| *Bos taurus* | HSPA5 | 93 | 1102 | 0.129 | 5 | 12 | 0.0047 | 64.2 | 0.528 | 0.398 | 4 |
| *Oryctologus cuniculis* | HSPA5 | 54 | 528 | 0.184 | 5 | 10 | 0.0086 | 33.8 | 0.597 | 0.334 | 4 |
| *Gallus gallus* | HSPA5 | 29 | 252 | 0.310 | 4 | 9 | 0.0199 | 12.0 | 0.646 | 0.273 | 2 |
| *Danio rerio* | Hspa5 | 97 | 1058 | 0.114 | 7 | 11 | 0.0042 | 76.9 | 0.465 | 0.371 | 5 |
| *Drosophila melanogaster* | Hsc70-3 | 57 | 760 | 0.238 | 5 | 14 | 0.0089 | 29.9 | 0.560 | 0.324 | 3 |
| *Caenorhabditis elegans* | hsp-90 | 109 | 1212 | 0.103 | 7 | 11 | 0.0034 | 98.9 | 0.594 | 0.450 | 6 |
| *Saccharomyces cerevisiae* | KAR2 | 150 | 2834 | 0.127 | 6 | 19 | 0.0028 | 108.0 | 0.607 | 0.352 | 6 |
| *Arabidopsis thaliana* | BIP2; BIP3 | 62 | 1188 | 0.314 | 5 | 19 | 0.0092 | 25.6 | 0.680 | 0.245 | 3 |


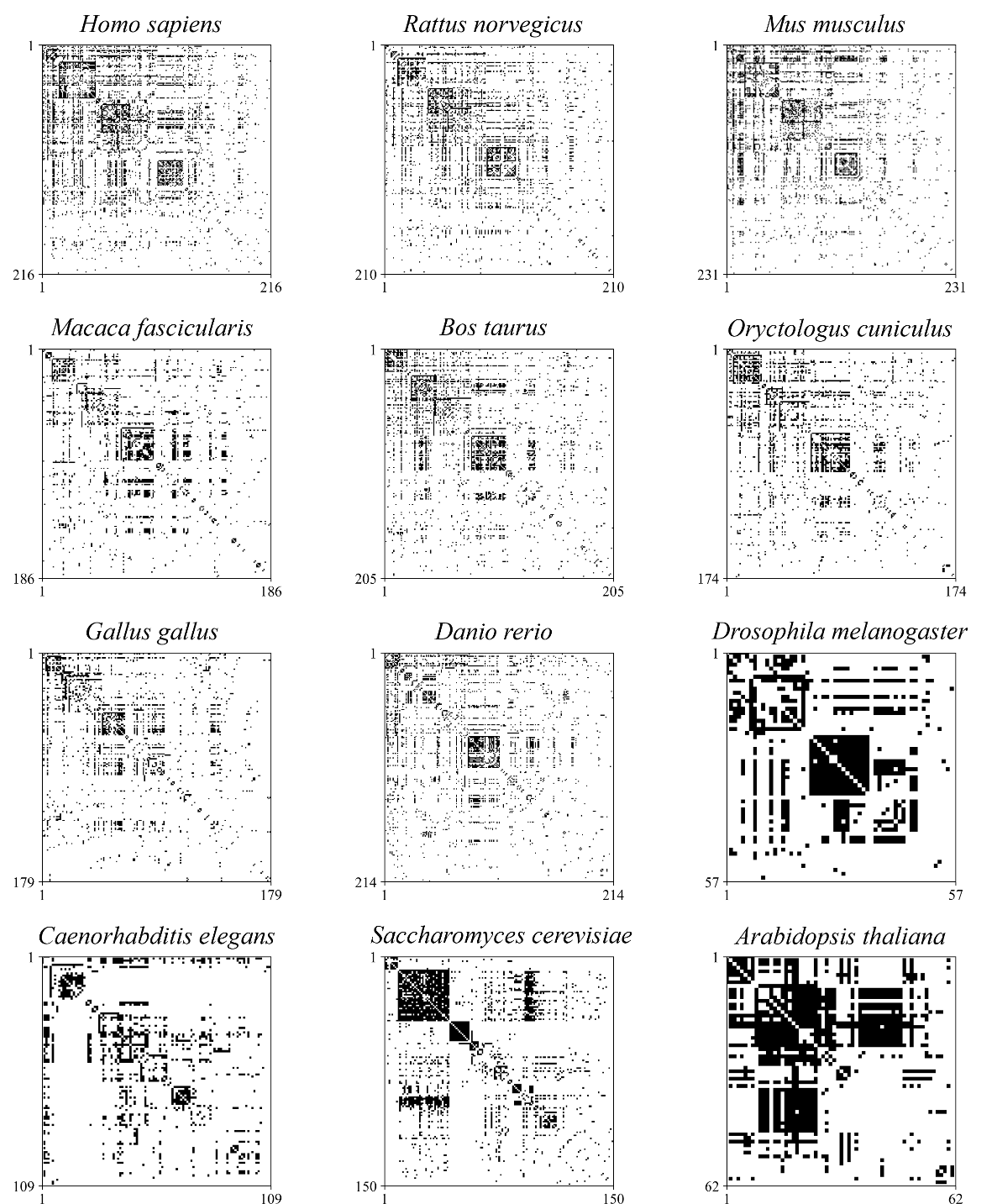


Fig. S1: Matrix representation of native models. Black dots in the matrix indicate the presence of an interaction between two proteins, while the withe elements are related to the absence of an interaction. The numbers on the axes identify the protein index within the network..


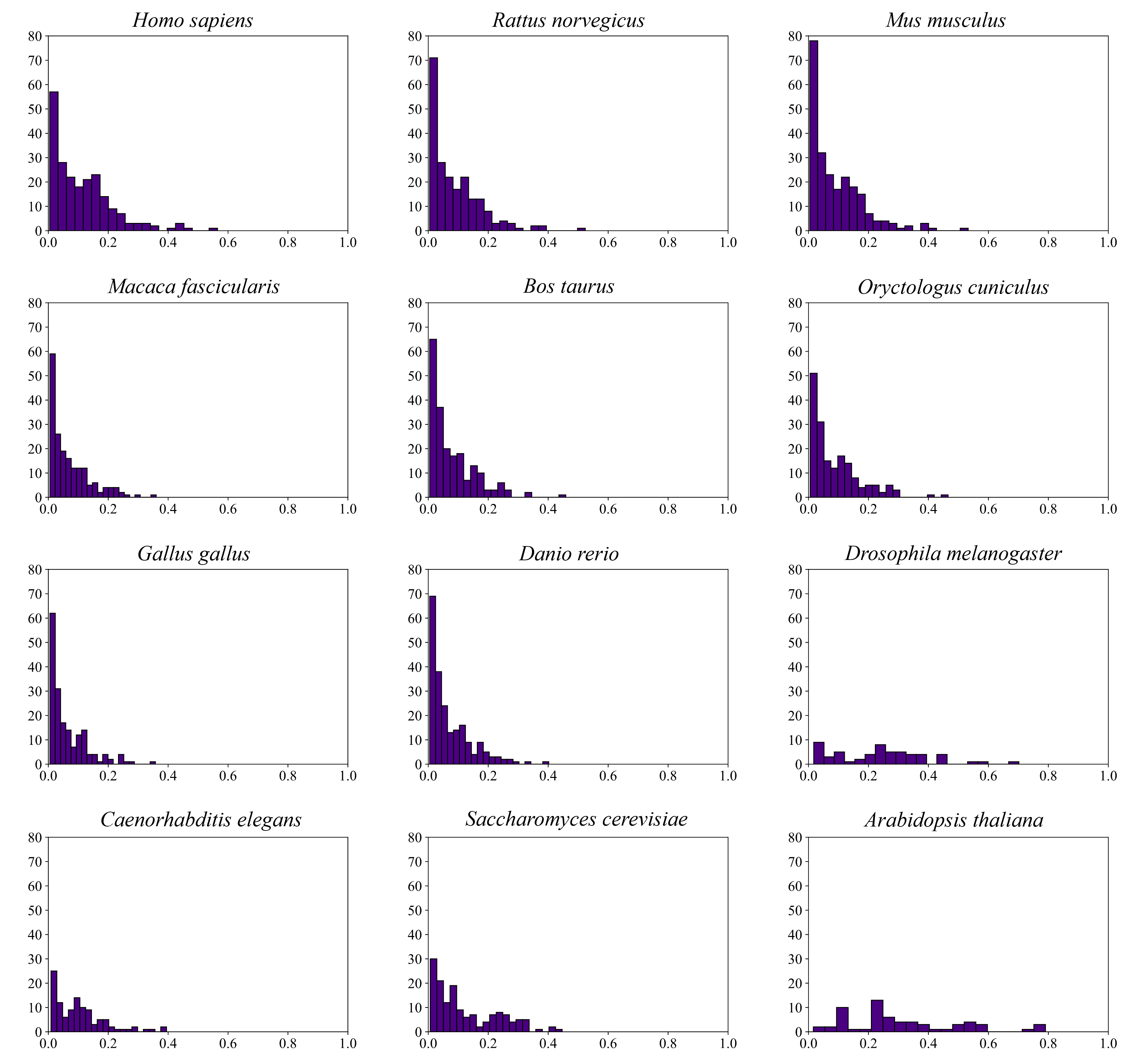


Fig. S2: Normalized node-degree distributions of native models.

**
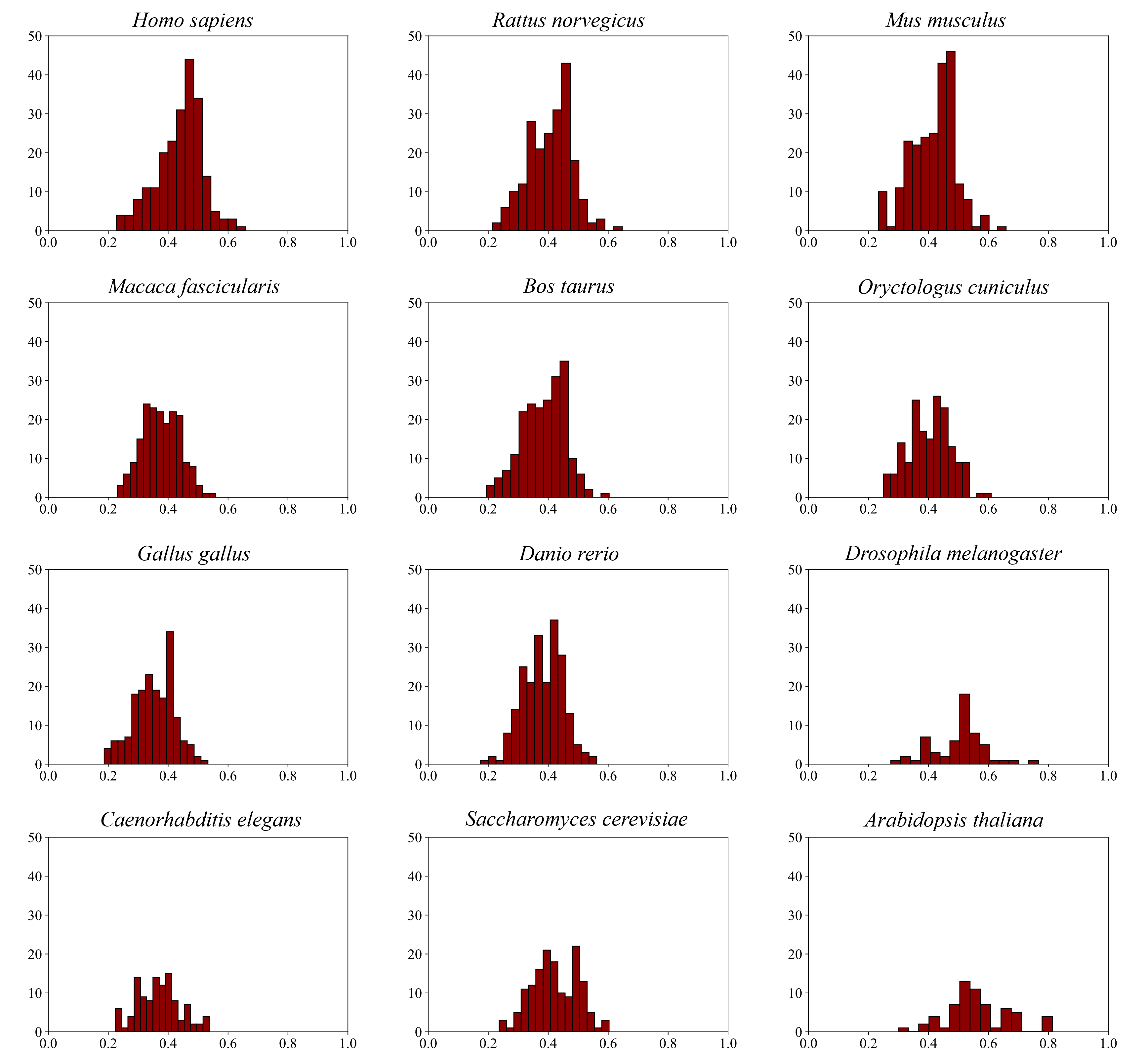
**

Fig. S3: Normalized closeness centrality distributions of native models.

Fig. S4: Normalized betweenness centrality distributions of native models.

**
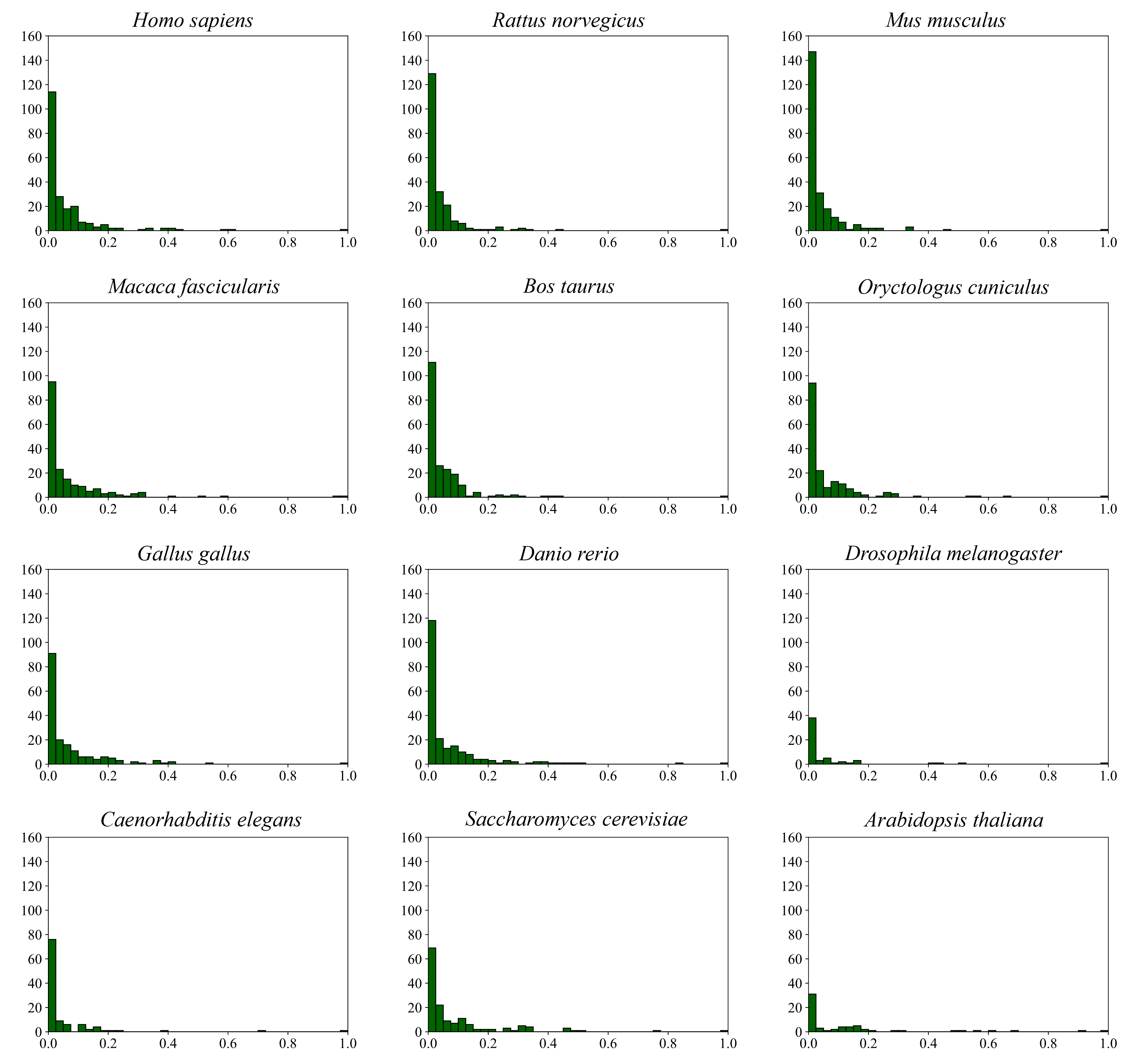
**


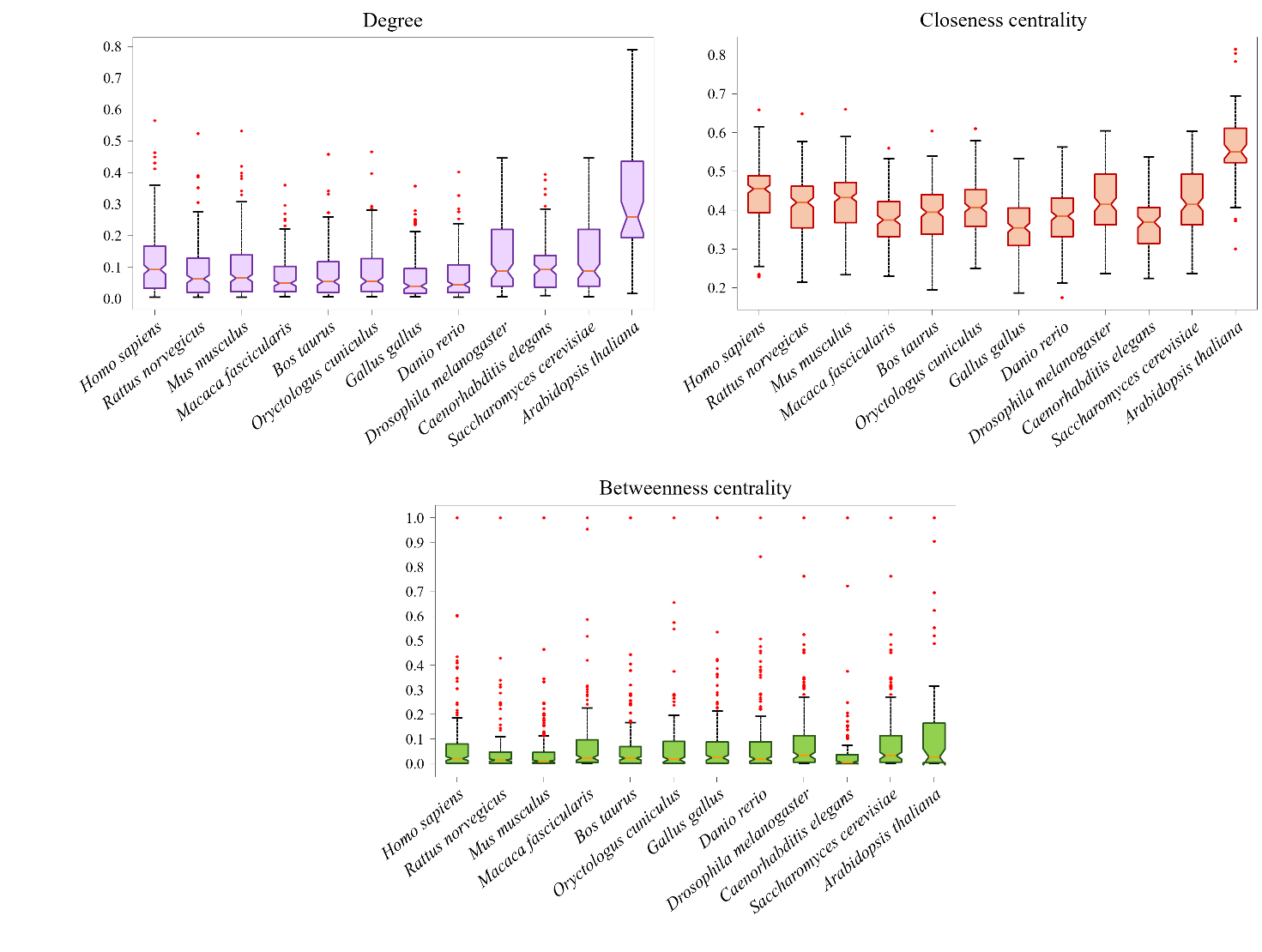


Fig. S5: Representation of normalized metrics distributions. Orange lines identify the median and red dots represent outlier points.


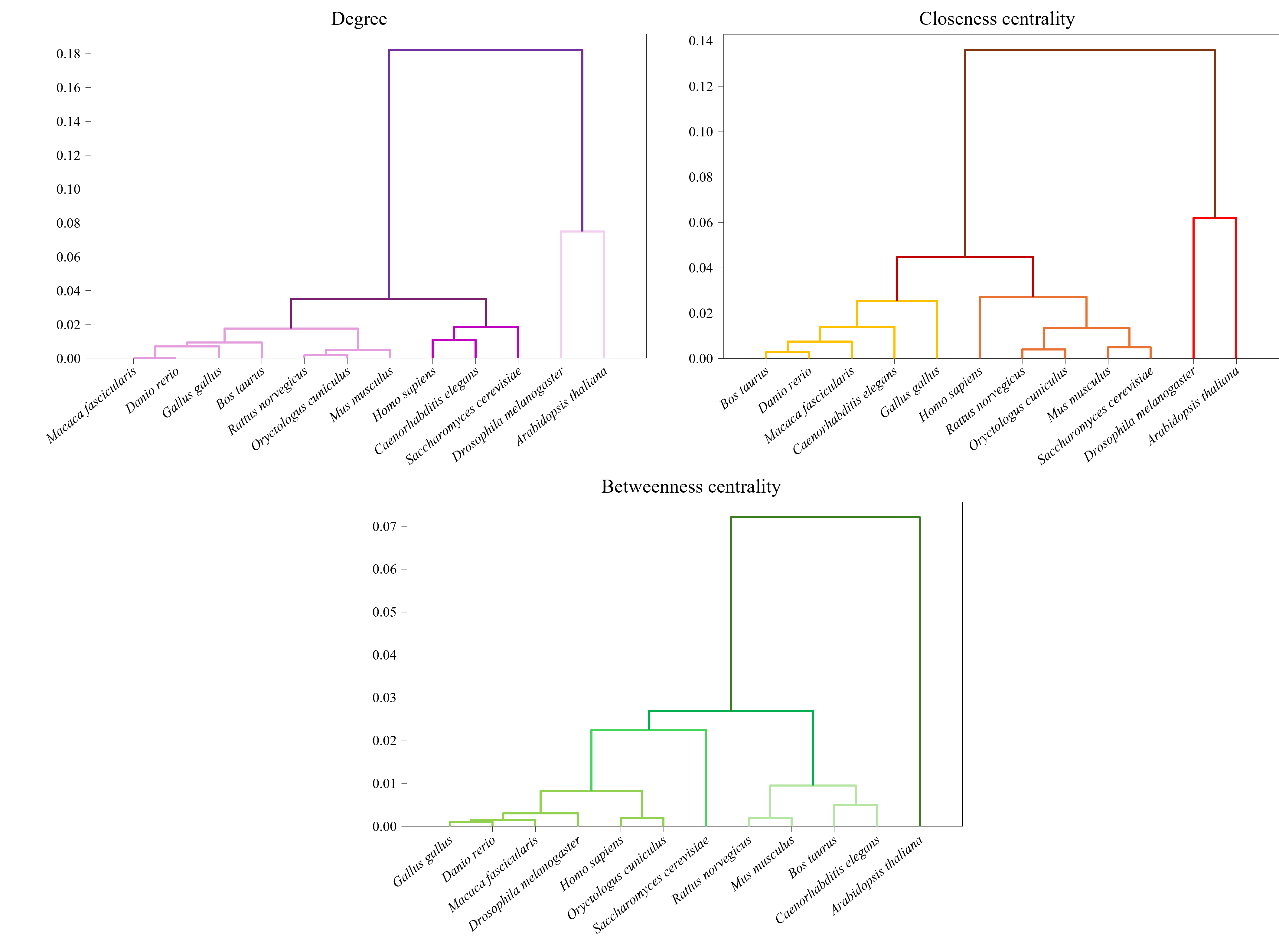


Fig. S6: Dendrogram tree of normalized average metric values. The hierarchical binary cluster tree is generated using the UPGMA for computing cluster distances.


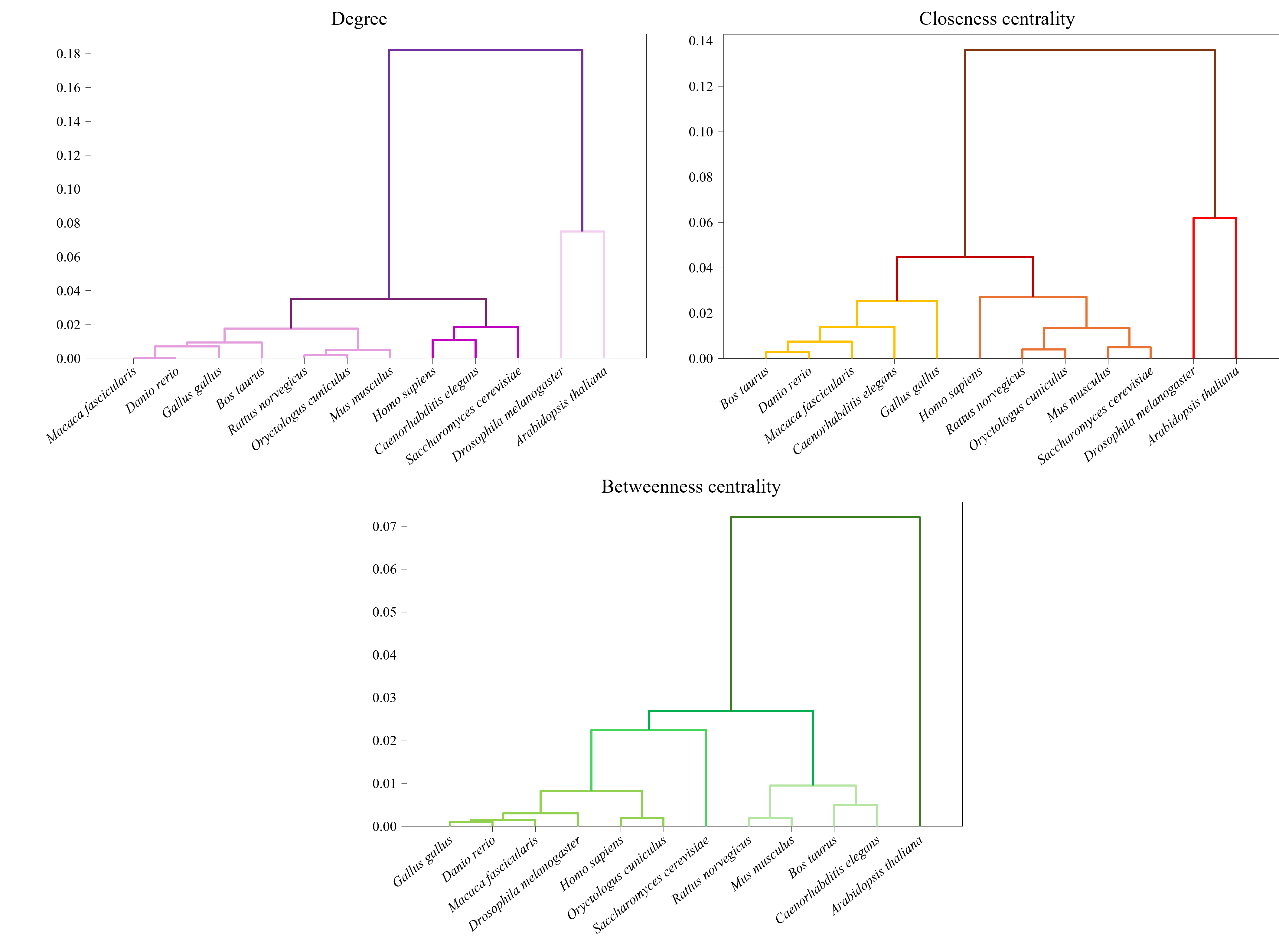


Fig. S7: Dendrogram tree of normalized metric medians. The hierarchical binary cluster tree is generated using the UPGMA for computing cluster distances.


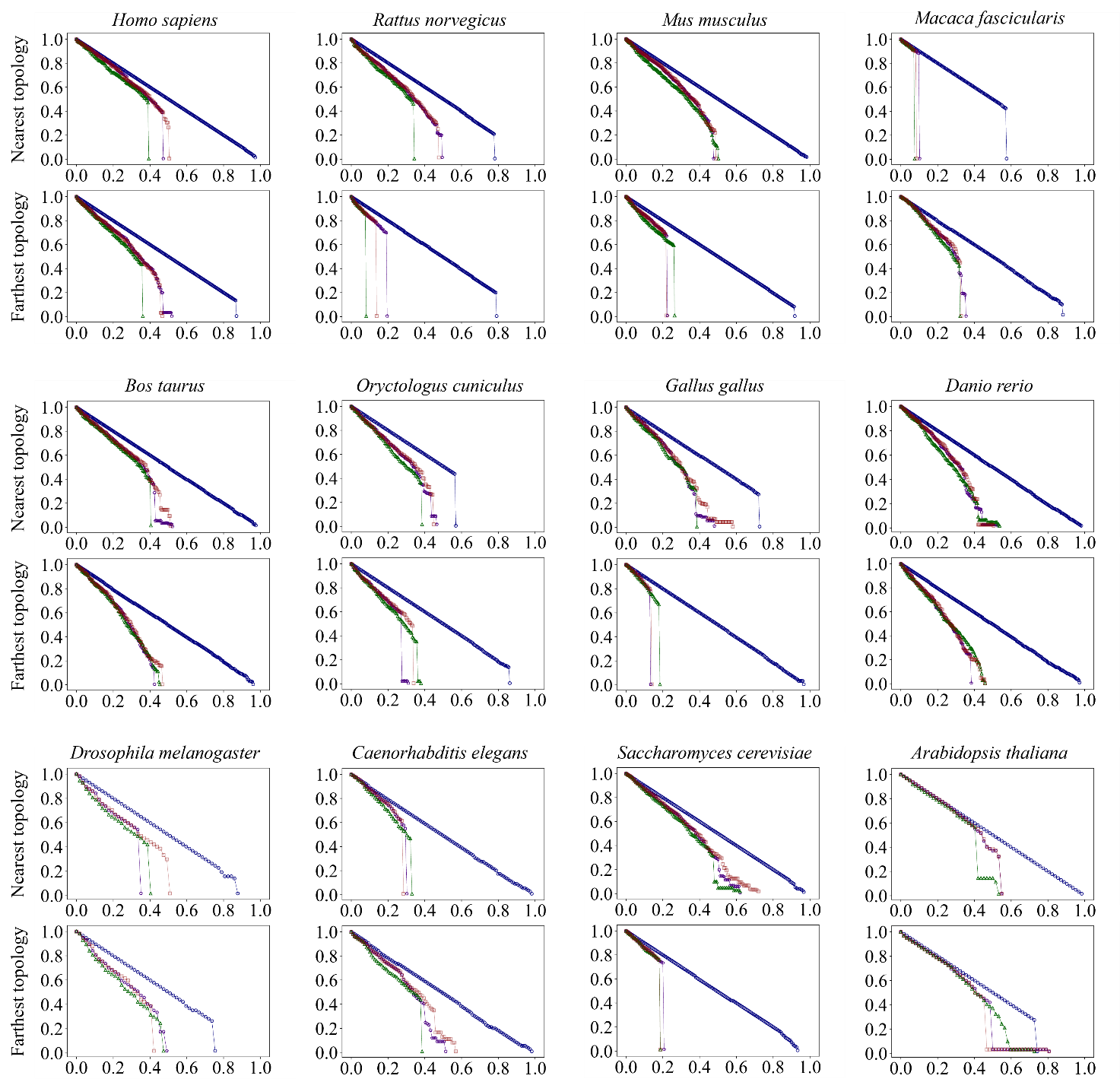


Fig. S8: Network robustness evaluated using random and targeted attacks on two configurational models (upper panels: nearest topology $-$ lowest dGHD, lower panels: farthest topology $-$ highest dGHD). Different colors identify attack strategy trends, same as native models (Fig. 5).
